# On Automatic Conversion from E-born PDF into Accessible EPUB3 and Audio-Embedded HTML5

**DOI:** 10.1007/978-3-030-58796-3_48

**Published:** 2020-08-10

**Authors:** Masakazu Suzuki, Katsuhito Yamaguchi

**Affiliations:** 8 Institute Integriert Studieren, JKU Linz, Linz, Austria; 9 Jack Baskin School of Engineering, UC Santa Cruz, Santa Cruz, CA USA; 10 Dipartimento di Meccanica, Politecnico di Milano, Milan, Italy; 11 Support Centre for Students with Special Needs, Masaryk University Brno, Brno, Czech Republic; 12 Institute of Mathematics for Industry, Kyushu University, Fukuoka, Japan; 13 Junior College Funabashi Campus, Nihon University, Tokyo, Japan

**Keywords:** E-born PDF, Conversion, Accessible e-books

## Abstract

As a promising method to make digital STEM books in PDF accessible, a new assistive technology to convert inaccessible PDF into accessible digital books in some different-type formats are shown. E-born PDF is initially converted into text-based EPUB3, and then, it is converted into audio-embedded HTML5 with JavaScript (ChattyBook). In the conversion, various local languages can be chosen for reading out STEM contents.

## Introduction

“Adobe Portable Document Format (PDF)” is commonly used for the exchange of STEM (science, technology, engineering and math) contents among researchers or in various educational fields. Although the PDF/UA standard is suggested as so-called “accessible PDF” [1], it is not necessarily easy to produce a document so as to conforms to the standard of PDF/UA. Unfortunately, most of PDF documents in the world are not accessible, and it is difficult to convert a given (inaccessible) PDF into PDF/UA automatically with a concise procedure. Furthermore, it should be emphasized that there are no well-established standards for an accessible STEM document including mathematical expressions in PDF.

In most cases, when considering PDF accessibility, target disabled people are usually assumed as the blind, and the conversion of PDF into Braille or text files with  notation has been mainly treated. However, there are many dyslexic people in the print-disabled, who can read neither a Braille document nor texts in the  notation. A large number of the low vision or people who have lost their sight in not-younger days cannot, either.

As a promising method to make digital STEM books in PDF accessible, here, we show a new assistive technology to convert inaccessible PDF into accessible digital books in some different-type formats. In the next section, we classify PDF into two categories, “E-born PDF” and “image PDF.” In this paper, we give a new tool to convert E-born PDF into text-based EPUB3 (the latest release of an open-ebook-standard EPUB), and then, into audio-embedded HTML5 with JavaScript (“ChattyBook”), so that the document content including math expressions is read out without a screen reader. In the conversion, various local languages can be chosen for reading out STEM contents.

## PDF Classification

To make our task much clearer, we begin with discussing different types of PDF. Nowadays, a PDF file is commonly produced from a digital format such as a document in Microsoft Word,  , Adobe InDesign, etc. In those PDF, the information on each character/symbol such as its character code, font type, coordinates on a page is usually embedded. You can cut and paste text information from them. As far as a math expression is concerned, its structure cannot be maintained through cut-and-pasting, but actually, its accurate character information can be detected even in the math expression by making use of a PDF parser. Based on the information, we can analyze its structure without an OCR (optical character recognition) process. We refer to such PDF as “E-born PDF” or simply, “ePDF”.

On the other hand, recently, image files are usually provided in PDF, which are made by scanning or copying. In principle, such PDF has no character information. Actually, many of them include not only images but also text information (a recognition result by OCR) in the background. However, it often includes a lot of recognition errors. Concerning a math part, you cannot use such background information to analyze the math structure since it usually consists of meaningless characters/symbols. We call them “image PDF”.

In some E-born PDF, to keep original layout in various display environment, characters/symbols are replaced with their scalable-vector (outline) images. In zooming-up, characters should be kept being fine, no matter how large they are magnified; however, the character information is not embedded in it. We call them “outline PDF”. Although they are actually E-born PDF, from the viewpoint of recognition processing, we classify them as a kind of image PDF.

## Accessible EPUB3

In this section, we discuss the conversion from E-born PDF into accessible EPUB3 [2]. There are several versions in EPUB3, and some of them are not compatible with each other. In this paper, we treat just EPUB3.1 which is expected to be popularized from now on. Here, we refer to it as EPUB3 or simply, EPUB.

There are two types of accessible EPUB. One of them includes audio files of aloud-reading as media-overlay, and the other does not. We call them “audio-embedded EPUB” and “text-based EPUB,” respectively.

In Japan, “the Japanese Society for Rehabilitation of Persons with Disabilities” has been providing print-disabled students with e-textbooks in multimedia DAISY (Digital Accessible Information System) format [3, 4]. They are now preparing to change the format of accessible e-textbooks to audio-embedded EPUB in the near future. In Japanese, four different character sets are used simultaneously in print: Chinese characters, Hiragana, Katakana and alphanumeric letters. While Hiragana and Katakana are essentially kinds of phonetic symbols, a single Chinese character or a compound of the characters usually has several ways of pronouncing, according to its context. In STEM, they are often read in a different manner from the usual. As the result, text-to-speech (TTS) engine tends to make mistakes quite frequently in reading out Japanese texts, and we do need to embed audio files of aloud-reading corrected manually in advance.

On the other hand, a TTS engine seldom makes such mistakes in English-speaking countries, and text-based EPUB is mainstream. It is possible to adopt a workflow to produce text-based EPUB initially and then, to convert it into audio-embedded EPUB. Thus, as the first step, we have developed a tool to convert E-born PDF into text-based EPUB.

## Conversion from E-born PDF into Text-Based EPUB

As was pointed out, in E-born PDF, the structure of math formulas cannot be maintained through cut-and-pasting. However, even in their inside, the accurate information on each character/symbol such as its character code, font type, coordinates on a page is embedded.

In the latest version of our OCR software for STEM, “InftyReader” [5], by making use of “vector-image information” for printing characters/symbols, which is provided by a powerful PDF parser, we can get not only character information but the true graphical area of the original character image even in the inside of mathematical formulas. Thus, it does not need any commercial OCR engines for recognizing/analyzing STEM contents in E-born PDF. Since character codes are precisely obtained, accurate conversion into text and mathematical-structure analysis can be done [6]. Furthermore, document structures such as chapters, sections, headings can be also obtained by classifying fonts used in the PDF. Thus, we have recently implemented a new function in InftyReader so that it can convert the recognition result of E-born PDF directly into text-based EPUB, in which math expressions are represented in MathML.

## Remaining Tasks

One of our remaining important tasks in STEM-document recognition is the segmentation of pages into figures/charts/diagrams/tables and main text areas (including math formulas). It is still difficult to realize the correct segmentation for a complicated-layout document. Analyzing the structure of tables including connected or multiple-line cells is also another remaining task. While simple tables are usually all right, InftyReader often fails at analyzing complicated-layout tables. Analyzing reading order of text areas is also difficult. As is well known, an order, in which text blocks are stored in the inside of PDF, is often different from the actual reading order. While analyzing the text order is not so difficult for a simple-layout PDF, recent school textbooks tend to have very complicated layout, and automatic text-order analysis is also a remaining important task.

In the ICCHP conference, we will show a tentative method to treat those tasks effectively, for the present. We developed an interface for InftyReader to allow users to correct manually area segmentation and their attributes including the reading order before the EPUB conversion.

## Audio-Embedded HTML5: ChattyBook

Recently, multimedia DAISY is widely accepted as a standard of accessible e-books for various print-disabled people such as the blind, the dyslexic, etc. Accessible EPUB is essentially DAISY, Ver.4 that does not exist as a DAISY version. However, we must confess that there also remain some problems to deserve greater attention even now; that is, ordinary DAISY contents and players are not necessarily useful enough for the students with dyslexia. For instance, unlike visually disabled people, they usually do not use a screen reader, and a good TTS engine is not installed in their computers, either. Furthermore, their environment such as devices, OS, players, etc. is different from each other. You have to customize the DAISY contents frequently to meet each user’s demands/environment.

To make DAISY/accessible EPUB be more useful for all, we developed a Windows application named “ChattyBooks” which converts DAISY/accessible-EPUB STEM content into audio-embedded HTML5 with JavaScript (ChattyBook) [7]. It consists of two component modules: a converter and a file manager. If a DAISY/accessible EPUB content is dropped on the ChattyBooks icon or in the ChattyBooks main window, it is converted automatically into HTML5 with JavaScript (a ChattyBook), and it is listed on the main window (bookcase) of ChattyBooks. When double-clicking a title on the bookcase, a browser such as Google Chrome displays the content which has the almost-same functionality and operability of high-quality as the original DAISY/accessible EPUB. An advantage of this scheme is that the converted book, ChattyBook, is an HTML5 document and can be played with any standard browsers, such as Google Chrome, Firefox, Edge, Safari, etc.

ChattyBooks uses Microsoft Speech API, Ver.5 (SAPI5) as a TTS engine. If multiple SAPI5 voices are available, a user can choose any of them in the conversion. Even if the original accessible-EPUB is text-based, in ChattyBook, aloud-reading of the entire content including technical notations such as math expressions is embedded as mp3 audio files, and users can listen to it in a high-quality voice with text/math highlighting to aid their comprehension even if they do not have a good TTS engine for themselves. In addition, since it is just HTML5 with JavaScript, it can be played by any popular browsers. User need not to use any DAISY/EPUB player. Disabled users can access easily the contents with their own environment: Windows, Mac, iPad, iPhone, Android, Chrome book, etc.

## Localization

In the latest version of our accessible STEM-document editor, “ChattyInfty3,” a new localization scheme has been compiled [8]. It allows end-users to incorporate the necessary definition files for aloud reading of mathematical notations in each local language efficiently/systematically into ChattyInfty3. The users can customize the software simply by putting the definition files in a specified folder and changing some software settings; then, ChattyInfty contents including math expressions are read out in that local language.

Actually, several local-language groups/individuals have been working on developing their own language versions: Czech, French, German, Greek, Italian, Kannada, Spanish, Turkish and Vietnamese versions of ChattyInfty3, most of which have been done without our help. It shows that ChattyInfty3 is actually customizable for various local languages by making use of the localization scheme. We have recently improved InftyReader so that the localization scheme is also available in converting text-based EPUB into ChattyBook. Thus, if the necessary definition files were prepared, a user could produce an accessible STEM book in their own local language easily from E-born PDF.

## Manner of Aloud Reading for Math Expressions

“What way of aloud reading is appropriate to access math expressions” depends on the user’s characteristic; each one has their own needs. While there is no problem in reading out a simple math formula, concerning a complicated/long math expression, one should figure out what way is appropriate to make it easy to understand.

In the conversion of text-based EPUB into ChattyBook, you can choose three types of aloud-reading for math formulas. “Plain Reading” is based on one which may be most widely used in English-speaking countries (the English version). It is natural, but a spoken mathematical expression is often ambiguous just only with speech. It is assumed that people with low vision and dyslexia use it. In “Smooth Reading,” minimum-necessary speech guides for blind users to grasp correctly the structure of a mathematical formula are added. “Detailed Reading” is assumed to be used when a blind user wants to know the mathematical-formula structure in the most detail.

In addition, we have also implemented a function in the application to control a time interval between math symbols or poses before/after them to make math expressions become easier to understand. Using ChattyBook, we are now planning evaluation by various print-disabled people to see which way is better for them.

## Further Tasks in the Future

The accessibility of school textbooks is probably most important in education. As was mentioned, in Japan, the Japanese Society for Rehabilitation of Persons with Disabilities has been providing print-disabled students with accessible e-textbooks in multimedia DAISY since 2008 [3]. They produced the greater part of requested textbooks for elementary and junior-high school (291 titles in 2018), and those textbooks were provided to more-than-10,000 print-disabled students (mostly ones with developmental reading disorder).

However, concerning senior-high school or higher education, the number of textbook titles is too large, and the same service is almost impossible (more-than-1,000 titles of senior-high school textbooks are published in Japan). Instead, we can expect that senior-high-school/university students could accept automatically produced textbooks in text-based EPUB. Even if a TTS engine would fail at reading out the content, they should be able to amend those errors mentally for themselves according to the context. If so, it might be possible for us to give a quick service of making a book accessible by automatically converting E-born PDF into text-based EPUB. (In Japan, it is regulated by law that the school textbooks in E-born PDF are provided by their publishers to organizations making them accessible for students with print disabilities.)

To realize that, however, layout-analysis technologies should progress remarkably to treat complicated-layout textbooks. In Japan, the layout tends to become more and more complicated; a lot of icons, illustrations, balloons and others appear quite frequently even in high-school textbooks. The most promising approach to analyze such complicated-layout document is machine learning. To use the machine-learning technology effectively, we do need a large quantity of annotated data for the learning.
